# On-Demand Scheduling of Command and Responses for Low-Power Multihop Wireless Networks †

**DOI:** 10.3390/s21030738

**Published:** 2021-01-22

**Authors:** Mingyu Park, Jeongyeup Paek

**Affiliations:** Department of Computer Science and Engineering, Chung-Ang University, Seoul 06947, Korea; hello0922@cau.ac.kr

**Keywords:** low-power and lossy network (LLN), multihop wireless network, dissemination and collection, resource allocation

## Abstract

Many IoT applications require a mechanism to disseminate commands and collect responses over a wireless network in order to control and collect data from multiple embedded devices. However, severe collisions may occur if a large number of nodes attempt to respond simultaneously and promptly, not only among the responses, but also with the dissemination of commands. This is because low-power wireless network protocols for dissemination and collection have been designed separately. Tuning the parameters of one side of the protocol has clear trade-off between reliability and latency. To address this challenge, we propose SCoRe, an on-demand scheme for joint scheduling of command and responses on multihop low-power wireless networks to improve both reliability and latency simultaneously at runtime. SCoRe gathers the amount of time required by network nodes for dissemination and collection, and allocates relative timeslots to each node recursively over multihop on-demand when (and only when) disseminating a command. While doing so, information exchange occurs only between local neighbor nodes without a need for global routing table nor time synchronization. We implement SCoRe on a low-power embedded platform, and compare with well-known dissemination and collection schemes through both simulations and testbed experiments on 30 devices. Our evaluation results show that SCoRe can improve both latency and reliability without tuning the parameters for one metric, while the legacy schemes require careful parameter selection to match only one side of SCoRe, never both.

## 1. Introduction

Emerging Internet of Things (IoT) technology is being applied to a variety of fields such as smart factory [1], smart grid AMI [2,3], smart market [4,5], and smart hospitals [6]. Most such IoT applications require a mechanism to control and collect data from multiple embedded devices deployed in the field of interest. For this purpose, they usually employ wireless networks to disseminate commands and receive responses, possibly over multihop [1,2,3,4,5,6,7,8]. However, if a large number of nodes respond simultaneously over a wireless network, severe collisions may occur—a phenomenon well known as the “ACK/feedback implosion” problem [9,10,11]. Furthermore, if the nodes respond promptly upon reception of a command, the responses will also interfere with the dissemination of commands. On the other hand, if the nodes respond slowly (e.g., wait for some time for command dissemination to hopefully finish), then the responses will be delayed, increasing the overall latency of the system. This problem can be more critical on applications that have large numbers of devices or are latency/loss sensitive.

There are a number of prior works that attempt to mitigate congestion in low-power wireless networks in order to improve reliability and/or latency. However, most of them focus only on one side of the problem: they either tried to improve either the downward command dissemination phase [12,13,14,15,16,17,18,19,20,21,22] or the upward response collection phase [23,24,25,26,27,28,29,30,31]. As both command-phase packets and response-phase packets collide not only among themselves, but also with each other, focusing only on one side is not enough for sufficient performance; they must be considered “jointly” and in parallel for real-world IoT applications.

To this end, we propose SCoRe, an “on-demand scheme for joint Scheduling of Command and Responses” on multihop low-power wireless networks to improve both reliability and latency simultaneously. SCoRe assigns dedicated “relative” transmission timeslots to each node to completely eliminate packet collisions. (Later in Section 4.3, we develop a concurrent/parallel transmission feature based on spatial reuse for improved latency.) To do this, SCoRe gathers “the number of desired timeslots for both command dissemination and response collection” ($N_{\mathrm{dslot}}$) from each node in the network. Then, upon reception of a command, SCoRe allocates subtree-aggregate timeslots to each next hop child node according to the their requirements recursively, layer-by-layer, as the command disseminates. Each node will transmit a response (if any) and re-disseminate the command message according to the assigned relative schedule. As a new relative schedule is calculated on-demand for each command and assigned asynchronously without time synchronization, SCoRe is adaptive to routing topology changes with minimal overhead. SCoRe is a cross-layer scheme that requires information from the network layer (i.e., route and hop info) and controls the transmission time at the link layer, and it can be used by IoT applications to resolve collision across “multihop” low-power wireless networks.

We implement SCoRe on a low-power embedded platform [32], and evaluate it through both real-world testbed experiments and simulations with 30 embedded devices and 1 gateway on various topologies. Our evaluation results show that SCoRe improves both round trip time (RTT) and packet reception ratio (PRR) with little packet retransmissions on all tested scenarios without tuning the parameters for one metric nor one topology. Parameters of the legacy schemes can be configured to match either the PRR or RTT of SCoRe, but never both. SCoRe achieves this by scheduling command and responses jointly.

The contributions of this work are as follows.

- Identify and demonstrate the problem of severe collisions between command dissemination and response collection because they were not considered “jointly”.
- Design SCoRe, an “on-demand scheme for joint Scheduling of Command and Responses”, on multihop low-power wireless networks to improve both reliability and latency simultaneously.
- Implement SCoRe on a real low-power embedded platform, and evaluate it through simulations and testbed experiments on 31 devices.

The remainder of this paper is organized as follows. Section 2 discusses the related work in the literature, and Section 3 motivates our work by showing why it is necessary to consider both command dissemination and response collection phases “jointly”. Then, Section 4 describes our proposed design of SCoRe, and Section 5 presents the evaluation results. We discuss the potentials of SCoRe in Section 6, and conclude the paper in Section 7.

## 2. Related Work

As the IoT technology pervades our everyday life, vast amount of research have been devoted to improve the performance of low-power multihop wireless networks for IoT applications. A number of studies [12,13,14,15,16,17,18,19,20,21,22] proposed fast flooding schemes to disseminate messages into the whole network using various ideas such as exponential timer, time synchronization, capture effects, etc. There are also numerous prior work on efficient collection of data in multihop low-power networks [23,24,25,26,27,28,29,30,31]. Although they are all great work that improves performance for their respective scenarios, they only targeted one side of the system;: either the command dissemination phase or the response collection phase. However, many, if not most, real-world IoT applications usually require some mechanism to both send commands and collect responses [1,2,3,4,5,6,7,8]. In other words, it is very common that command and response packets coexist in the network concurrently in real-world IoT applications.

There are some studies which tried to schedule commands and responses jointly. Aijaz et al. [33] proposed DeAMON, a decentralized packet scheduling for 6TiSCH [34] in multihop wireless networks. DeAMON’s resource pool is partitioned into signaling, data, and over-provisioned slotframes. The root disseminates a Build command via flooding in a scheduled manner, upon which a receiving node will start scheduling itself. DeAMON’s scheduling guarantees parallel transmissions between far-away nodes by using Request-for-Slots and Assign messages. However, DeAMON uses one dedicated channel in 6TiSCH just for disseminating Build commands (control channel), and signaling, data, and overprovision are all operated on separated channels. Thus, it is not suitable for single-channel scenarios. In addition, DeAMON requires extra control packet overheads for scheduling. DeTAS (Accettura et al. [35]) and OST (Jeong et al. [36]) are also decentralized packet scheduling schemes for 6TiSCH/TSCH networks that address collision and congestion problems, but they focus only on either the collection phase or MAC layer and does not consider command dissemination phase jointly.

Voigt et al. [1] proposed CoReDac which schedules both command and response packets in a multihop network. CoReDac’s time slotting mechanism operates in the MAC layer, and scheduling is done via MAC layer packet exchanges between parent and child by piggybacking slot offset and sleep interval information into messages, whereas SCoRe’s scheduling is done at a higher layer and at a larger scale for the whole multihop network recursively. Furthermore, although CoReDac schedules command and response phase together similar to our approach, their evaluation focuses on energy-efficiency not reliability nor latency. For most IoT applications, especially industrial applications, reliability and latency are both crucial performance metrics that must be satisfied jointly.

H. Zhang et al. [37] proposed a latency-optimal convergecast scheme for WirelessHART network. WirelessHART standard supports channel hopping-based TDMA scheme, and the authors improve this in terms of latency and the number of channels used. However, WirelessHART employs proactive scheduling which requires time synchronization in contrast to our on-demand relative scheduling. Moreover, the root manages timeslots of each node one by one which incurs high overhead, while SCoRe uses recursive scheduling of subtree aggregate slots. In addition, the proposed scheme was evaluated via simulation only whereas we conduct experiments to demonstrate the effectiveness under real wireless environment.

## 3. Problem

We first investigate the performance problem when collection packets are (near) synchronized, and when command and response phase packets coexist. We implement a “Flooding + RPL -collection” (RPL is the IETF Internet standard IPv6 Routing Protocol for Low-power and Lossy Networks defined in RFC 6550 [24], popular in many IoT applications [38]). application scenario where commands are flooded into the network periodically from a root node, and each embedded node transmits a response to that command over multihop. When transmitting, each node will use random jitter ranges, $T_{C}$ for command dissemination and $T_{R}$ for response collection, to delay its transmission slightly. This is in an attempt to avoid synchronization between multiple receivers—the so-called “ACK/feedback-implosion” problem [9,10,11]. A node receiving a command forwards it and responds to it within $T_{C}$ and $T_{R}$ milliseconds, respectively, where the time is chosen uniform randomly. For the commands, a node transmits *M* times for reliability (Since there is no link-layer ACK for link broadcasts).

We implement this on TinyOS 2.1.2 [39], and conduct simulations using Cooja simulator [40]. We use BLIP and TinyRPL [41] in TinyOS as the UDP/IPv6 stack and RPL implementation, respectively. Each node is a TelosB [32] with an MSP430 microcontroller and a CC2420 radio, and communicate with each other over IEEE 802.15.4 links with CSMA.

**Figure 1:**
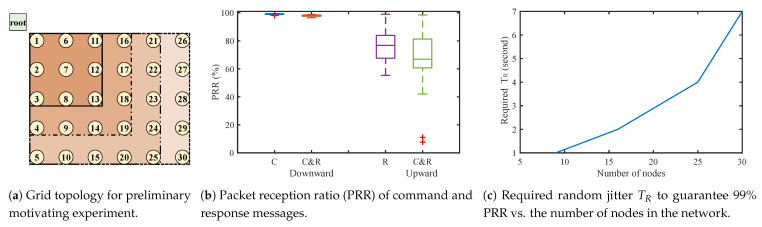
Grid topology for the preliminary simulation and the result.

With this implementation, we run simulations for three cases: “command dissemination only” (C), “response collection only” (R), and “command and response together” (CR) on grid topology as shown in Figure 1a. $T_{R}$ and $T_{C}$ are set to 1000 ms and 200 ms, respectively, and the number of command broadcasts *M* is 3. The root generates 1000 command packets, and every node responds with a message for every command. All messages are UDP over IPv6. Figure 1b,c plot the results of our preliminary experiment.

Figure 1b shows that downward (from root to embedded node) PRR is much better than upward (from embedded node to root) PRR, and upward PRR performance worsens significantly when command packets exist; downward PRRs are almost 99% in both *C* and CR case, but upward PRRs is 76.7% in *R* case and CR is 66.5%. First part is because dissemination is based on link-layer broadcast, thus every node can receive commands from all neighbors not only their parents, whereas responses are sent via unicast through the routing paths established by the routing protocol. More importantly, when collecting, 30 nodes are generating hundreds of link transmissions to travel over multihop within ∼1000 ms, i.e., severe link congestion, collisions, and queue overflows are occurring at bottleneck nodes. Command packets make things significantly worse (CR compared to *R* case). Therefore, scheduling both command and response phases in conjunction is essential, not optional, for network performance.

Next, we adjusted the parameter $T_{R}$ to investigate how much latency we would need to sacrifice in order to achieve 99% reliability. Figure 1c presents the required minimum random jitter $T_{R}$ to achieve 99% PRR as the number of network nodes is increased (with fixed $T_{C}$ = 200 ms). We ran simulations with 4, 9, 16, 25, and 30 nodes as presented in squares of Figure 1a. The required $T_{R}$ increases sharply as the number of nodes increases due to larger network depth and increased number of transmissions, as expected. This naturally leads to increased latency and higher response delay. The real challenge is, in real-world applications and systems, a network manager would need to carefully pre-tune the parameters (e.g., random jitter $T_{R}$) depending on the network size and topology to achieve its performance goals. We have learned this the hard way through several real-world implementations and deployments of IoT projects [4,5,6,7]. These facts necessitate a dynamic and adaptive mechanism to disseminate commands and receive responses in a multihop IoT network, without careful manual parameter tuning, to satisfy both reliability and low-latency requirements.

## 4. SCoRe Design

This section presents the design of SCoRe based on the following system requirements for joint packet scheduling in multihop wireless networks. SCoRe targets IoT application scenarios in which commands are disseminated into the network from the root (e.g., server, gateway, or access point), and their corresponding responses need to be collected reliably with low latency.

- Adaptation to network topology: Routing topology in wireless networks are rarely static, and any inconsistency between route and schedule may result in significant performance loss. Therefore, resource scheduling should dynamically adapt to number of devices, physical relocation, and routing topology changes possibly due to link quality variations.
- Little control/memory overhead: Low-power embedded systems with resource constrained devices are typically intolerant of extra packet overhead for energy and bandwidth reasons. Furthermore, multihop routing protocols may take either the storing mode or non-storing mode [24,42] approach depending on the memory constraints for routing tables. Thus, scheduling protocol should generate minimal packet overhead, information exchange should be done locally without a global routing table, and should support both storing and non-storing mode of operation. Furthermore, global time synchronization in a multihop network is a complex task [43,44] and should be avoided if possible.
- Efficient resource assignment over multihop: Because we target multihop, the number of total transmissions required to reach the root (even for same number of devices) depends on the location of each node in the topology. Assigning a dedicated, exclusive transmission slot within the whole network may be a must in a 1-hop TDMA system for fair channel access, but would be too naïve in multihop networks. Nodes that do not interfere with each other should be able to transmit concurrently (spatial re-use) for improved latency and bandwidth.

SCoRe is designed to satisfy these requirements while disseminating commands and collecting responses reliably and promptly. Each SCoRe node calculates “the number of desired timeslots for both command dissemination and response collection” (hereinafter referred to as $N_{\mathrm{dslot}}$) it needs based on its routing information. Each node also calculates its subtree-aggregate $N_{\mathrm{dslot}}$ (including those needed for its subtree), and reports that to its routing parent. Then, the sum of these $N_{\mathrm{dslot}}$ is piggybacked in regular routing messages [23,24], and will eventually reach the root. When a command needs to be disseminated, SCoRe’s root debriefs these $N_{\mathrm{dslot}}$ from network nodes, and assigns required aggregate timeslot chunks to (and only to) each of its 1-hop children. Then, upon reception of a new command, each node uses assigned slots for itself and its subtree nodes recursively. The details of SCoRe are explained in following subsections.

### 4.1. Recursive $N_{\mathrm{dslot}}$ Gathering

**Figure 2:**
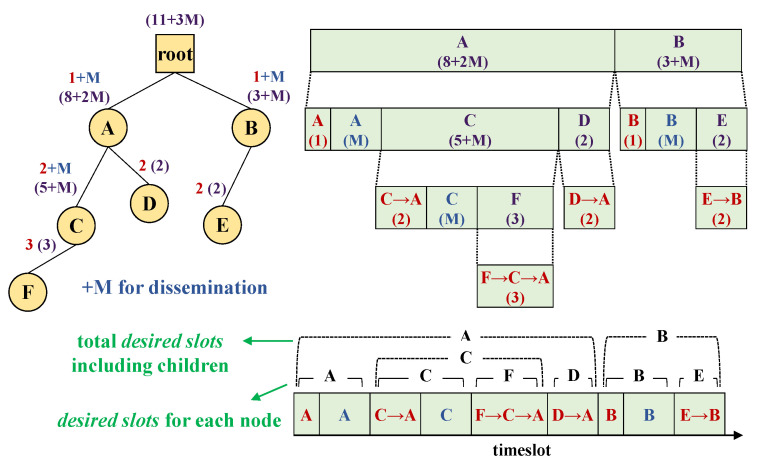
SCoRe’s scheduling process overview.

Figure 2 illustrates the key idea of SCoRe through an example. SCoRe allocates $N_{\mathrm{dslot}}$ to each node where $N_{\mathrm{dslot}}$ consist of two components: (1) $N_{\mathrm{dslot}}^{\mathrm{my}}$, which is the sum of response-phase slots and command-phase slots for itself, and (2) $N_{\mathrm{dslot}}^{\mathrm{subtree}}$, which is the sum of $N_{\mathrm{dslot}}$ of its subtree. $N_{\mathrm{dslot}}^{\mathrm{my}}$ for response-phase is the hop count *H* of each node (as a packet from this node requires *H* transmissions to reach the root), and *M* for command-phase which is the number of transmissions configured for command dissemination (e.g., flooding [13,22]). That is, $N_{\mathrm{dslot}}^{\mathrm{my}}$ = *H* + *M*. For example, $N_{\mathrm{dslot}}^{\mathrm{my}}$ for a 3-hop node is 3+*M*, 3 for response, and *M* for command. SCoRe does assume that it can obtain parent–child relationship and hop-depth information from its routing tree topology, which is common in most routing protocols for low-power wireless networks [23,24,45,46].

For SCoRe to gather $N_{\mathrm{dslot}}$ (2 bytes) from network nodes, each node informs its parent by piggybacking the sum of the number of timeslots it needs ($N_{\mathrm{dslot}}^{\mathrm{my}}$) together with an aggregate sum of $N_{\mathrm{dslot}}$ of its 1-hop children ($N_{\mathrm{dslot}}^{\mathrm{subtree}}$) in the upward route-notification packet such as DAO in RPL [24] (Without loss of generality, other similar routing protocols [23,46] can be used.). In Figure 2, for example, node A requires 1 + *M* slots for itself ($N_{\mathrm{dslot}}^{\mathrm{my}}$), and additional 7 + *M* slots for its subtree ($N_{\mathrm{dslot}}^{\mathrm{subtree}}$), totaling to 8 + 2M ($N_{\mathrm{dslot}}$). The parent receiving $N_{\mathrm{dslot}}$ from its children does not have to know how many $N_{\mathrm{dslot}}$ each of its descendants need; it just needs to know the total amount of $N_{\mathrm{dslot}}$ its subtree rooted at that child will use. This aggregated number will eventually reach the root recursively via regular routing updates (e.g., DIO/DAO in RPL) without creating extra packets.

Calculation of total $N_{\mathrm{dslot}}$ can be expressed as Equation (4),

(1) $$\begin{array}{cc} N_{{\mathrm{dslot}}_{i}}^{\mathrm{my}} & =H_{i}+M \end{array}$$

(2) $$\begin{array}{cc} N_{{\mathrm{dslot}}_{i}}^{\mathrm{subtree}} & =\sum_{j=1}^{L}N_{{\mathrm{dslot}}_{j}},\left(\mathrm{for}\;\forall j\in 1-\mathrm{hop}\;\mathrm{children}\;\mathrm{of}\;i\right) \end{array}$$

(3) $$\begin{array}{cc} N_{{\mathrm{dslot}}_{i}} & =N_{\mathrm{dslot}}^{\mathrm{my}}+N_{\mathrm{dslot}}^{\mathrm{subtree}} \end{array}$$

(4) $$\begin{array}{cc} \; & =\sum_{j=1}^{L}N_{{\mathrm{dslot}}_{j}}+H_{i}+M \end{array}$$

where *i* and *j* denote nodes that calculate total $N_{\mathrm{dslot}}$ and its child, respectively. *L* is the number of *i*’s children. *i* forwards the sum of its children’s $N_{\mathrm{dslot}}$, its hop count *H* and the number of dissemination *M* to its parent. *M* may be omitted for leaf nodes (if such information exists in the routing protocol) because leaf nodes need not be responsible for dissemination.

SCoRe’s gathering allows each node to know the total time needed for command dissemination and response collection in its sub-network, and thus it can schedule the (relative) transmission times in its subtree, on-demand when needed.

### 4.2. Recursive $N_{\mathrm{dslot}}$ Scheduling

Scheduling is similar to gathering, but needs one more scheme to avoid slot violations. SCoRe schedules relative transmission timeslots layer-by-layer based on $N_{\mathrm{dslot}}$ information obtained via recursive $N_{\mathrm{dslot}}$ gathering. When (and only when) disseminating a command, SCoRe root builds a relative time schedule with enough timeslots to cover the total amount of $N_{\mathrm{dslot}}$, and allocates timeslots to each 1-hop child according to their $N_{\mathrm{dslot}}$. The assigned timeslot information is carried into and disseminated via command messages, where 4 bytes is used per 1-hop child; 2 bytes is the IEEE 802.15.4 short address and 2 bytes for the allocated aggregate timeslot length. Then, every node will redistribute the assigned slots to its 1-hop children recursively.

A node receiving a command finds the relative timeslot offset (from the position of its own short address in the sequence) and length (2 bytes) assigned to itself in the message. In Figure 2, for example, suppose *M* is 1 and the routing tree has been established through which $N_{\mathrm{dslot}}$ has been gathered. SCoRe root knows that node A and B require 10 and 4 timeslots, respectively, thus it assigns timeslots according to their requirements sequentially. Node B knows that it can start its transmission (including its subtree members) after A’s reservation of 10 timeslots, and use 4 timeslots for itself and its subtree.

Each node uses a subset of the assigned timeslots to transmit its own response message (to the command), and also to forward commands. The remaining timeslots are rescheduled for its subtree nodes. For instance, node A in Figure 2 uses 2 timeslots from the 10 assigned to itself, and the remaining 8 timeslots are rescheduled, 6 for node C, and 2 for D.

**Figure 3:**
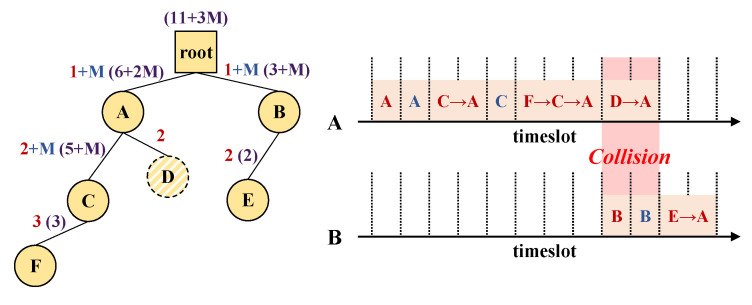
Slot violation scenario.

$N_{\mathrm{dslot}}$ scheduling is processed recursively similar to $N_{\mathrm{dslot}}$ gathering, but has a critical difference that a node should never use more than what it receives. Suppose node D has recently joined the network and the root is unaware of D’s $N_{\mathrm{dslot}}$ yet as shown in Figure 3. Node A’s $N_{\mathrm{dslot}}$ would have been 8 in the past, and thus the root will allocate only 8 slots for node A despite it needs 10 slots total to support D’s responses. If node A uses 2 more slots to cover node D, then the transmissions for delivering D’s response packet (D→A, A→root) will disrupt node B’s transmissions (response and dissemination) which will result in packet collisions. Therefore, node A should not allocate timeslots to node D in this case.

### 4.3. Concurrent Transmissions for Spatial Reuse

It is intuitive that assigning a dedicated, exclusive timeslot for each transmission within the whole multihop network may result in a very inefficient use of the wireless channel if the network is large enough for spatial reuse. For example, suppose we have a linear routing topology having only 1 child per each node for *h*-hops. The total number of $N_{\mathrm{dslot}}$ for this network can be expressed as

(5) ∑Ndslot=h(h+1)2︸∑Ndslotr+h(h+1)2(h−1)M︸∑Ndslotc

resulting in the worst case for command-response latency (The (h−1) in the last term assumes that the leaf nodes do not re-dissminate the command messages.).

**Figure 4:**
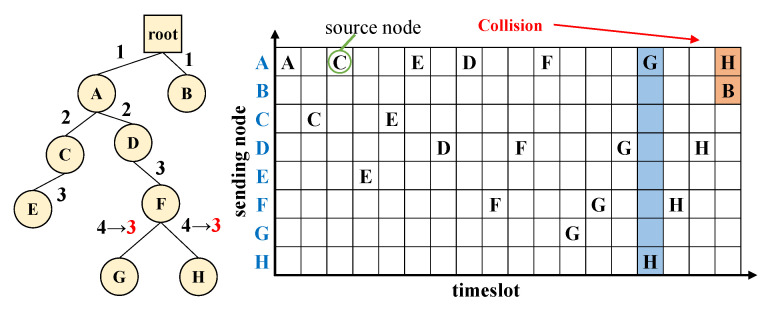
An example of parallel transmission scenario.

Nodes that are far away from each other should be allowed to transmit data concurrently for lower latency as long as they do not interfere with each other. For this purpose, SCoRe’s design allows concurrent transmissions assuming that 3-hop-away nodes do not interfere each other stochastically in hop-count-based RPL. This is based on the widely accepted intuition that maximum interference range of a wireless transmission is within twice the maximum communication distance [47]. For example, suppose a routing tree is built as shown in Figure 4. Node G requires 4 slots for its response, but it knows that its packet will reach 1-hop node A after 3 timeslots, and node A’s transmission is unlikely to interfere with node G or H’s transmission. Therefore, node H’s packet can coexist with those from node A. Thus, node G requires 3 response timeslots rather than 4, and node H may transmit a slot earlier with node A’s transmission. Using this idea, the total $N_{\mathrm{dslot}}$ for the worst-case linear topology reduces to

(6) $$\begin{array}{c} \begin{array}{cc} \sum {slot}_{\mathrm{res}}\; & =1+2+3+3+...+3\\ \; & =\left\{\begin{array}{cc} 4h-6 & \left(h>2\right)\\ \frac{h(h+1)}{2} & \left(h\leq 2\right) \end{array}\right. \end{array} \end{array}$$

On the other hand, node H’s transmission can disrupt a B’s packet if node H reserves 3 timeslots as shown in Figure 4. This timeslot compression scheme must guarantee inviolateness, but each node has no way of knowing which node sends after its transmission. However, the problem occurs only when a leaf node reduces its $N_{\mathrm{dslot}}$, and each node knows whether it has a child. In addition, each node can identify its relative timeslot order from command messages among neighbors having the same parent. Thus an SCoRe node reduces $N_{\mathrm{dslot}}$ if (1) it has a child, or (2) its timeslot is not located last in the list.

### 4.4. Faster $N_{\mathrm{dslot}}$ Updates

**Figure 5:**
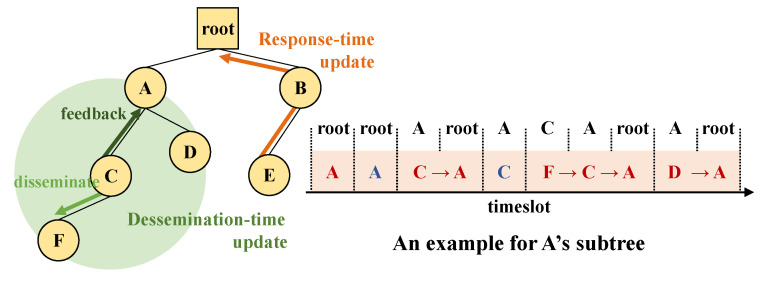
Response and dissemination time update.

Routing topology in wireless networks may change due to various reasons such as link fluctuations or node join/leave, and any inconsistency between route and schedule may result in significant performance loss. This problem will persist until SCoRe root receives updated and accurate $N_{\mathrm{dslot}}$ information, and since SCoRe rely on route control packets to propagate $N_{\mathrm{dslot}}$ information, its performance will depends on routing protocol’s behavior. Most routing protocols, however, try to send routing updates as few as possible to improve energy efficiency and reduce control packet overheads (e.g., Trickle timer for RPL’s DIO and DAO). Therefore, SCoRe requires other ways to respond to network changes as fast as possible. To detect and resolve this $N_{\mathrm{dslot}}$ inconsistency problem promptly, SCoRe employs two small but effective recovery techniques as shown in Figure 5.

**Response time update.** Because SCoRe’s timeslot scheduling is up to each parent and all response messages must pass through the parent of a sender, this is a great opportunity to resolve $N_{\mathrm{dslot}}$ inconsistency. An SCoRe node piggybacks its $N_{\mathrm{dslot}}$ in the response messages so that its parent can check/update its $N_{\mathrm{dslot}}$. When the parent forwards the message to its parent, it modifies the value of $N_{\mathrm{dslot}}$ field to its own demand. Through this recursive process, newly updated $N_{\mathrm{dslot}}$ information from a response source is aggregated and reaches the root at response time.
**Dissemination time update.** SCoRe’s command messages are based on link broadcast, and a parent also belongs to a child’s 1-hop neighbors. Thus, a parent is also able to hear the command messsage transmission from its child, although it is meant to go downwards. SCoRe uses this characteristic to update any $N_{\mathrm{dslot}}$ inconsistency. An SCoRe node embeds its $N_{\mathrm{dslot}}$ into command messages also, and its parent can overhear and update $N_{\mathrm{dslot}}$ of that node.

The overhead for these recovery schemes is just the size of $N_{\mathrm{dslot}}$ field, which is only 2 bytes in our implementation.

## 5. Evaluation

We evaluate SCoRe by comparing it to legacy protocols in terms of “packet reception ratio (PRR)”, command-to-response “latency”, and “number of retransmissions”. PRR is calculated as a product of downward PRR (command-phase) and upward PRR (response-phase). Latency is measured as the round trip time (RTT) from the transmission of a command to until the last successful response arrived at the command originator, the root. The number of packet retransmissions is averaged for response packets only as command dissemination is based on link broadcast. We conduct both experiments and simulations with 31 devices: 30 embedded nodes and 1 gateway (as a root).

### 5.1. Evaluation Setup

We use two protocol combinations for comparison to SCoRe: “Flooding + RPL” and “Trickle + RPL”, where Flooding and Trickle are used for command dissemination, and basic RPL is used for response collection.

“Flooding” is the most basic algorithm to disseminate information through a multihop wireless network. A node receiving a new (Sequence numbers are used to identify “new”.) message forwards that to its neighbors *M* times with random jitter $T_{C}$.

“Trickle” [13] is an efficient data dissemination algorithm, designed to propagate information faster when inconsistency is detected but with less overhead otherwise. A node receiving a message schedules the next transmission time from the range $\left[T_{C}/2,T_{C}\right)$. While waiting, the node counts the number of duplicates it receives. Then, when it is time for transmission, it transmits the packet if the number of duplicates is smaller than the specified suppression threshold *K*. Otherwise, it keeps silent for energy efficiency. Trickle doubles $T_{C}$ value after every retransmission, and resets to initial value when it receives a new packet or inconsistency is detected. Trickle algorithm has been adopted in various protocols for low-power wireless networks [7,18,22,24,48,49,50,51].

“RPL” is an IETF Internet standard IPv6 routing protocol for low-power and lossy networks (LLN) [24], and is used to collect data from nodes in multihop networks. In our application scenario, each RPL node sends its response packet with a random jitter $T_{R}$ (at the app layer, not routing layer).

All protocols are implemented using TinyOS 2.1.2 [39] on TelosB [32] platform (identical to Section 3) communicating over IEEE 802.15.4 links with CSMA. The network builds route topology according to the RPL protocol, and OF0 [52] is used as the objective function. The root starts transmitting commands 15 min after reset to allow for routes to stabilize, and generates 1000 commands every 5 s. Response packets are sent with a random time jitter in the range of $T_{R}$ milliseconds to avoid synchronization effect.

### 5.2. Parameter Selection for Legacy Schemes

**Figure 6:**
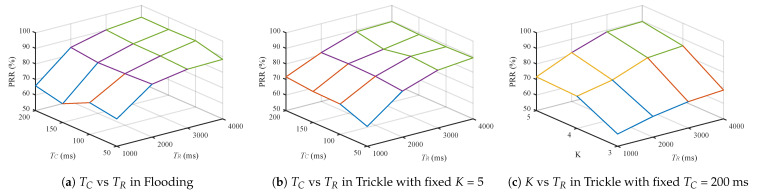
A 3D plots representing Packet Reception Ratio with varying parameters.

First, we conduct simulations while varying each parameter, $T_{C}$, $T_{R}$, and *K* to find appropriate values for each algorithm in our setup. Figure 6 plots the influences of each parameter. In the simulations, we set *M* to 3 to cope with unexpected losses (e.g., inter-node interference/collision or queue losses). Figure 6a,b show that PRR is little influenced by dissemination random jitter $T_{C}$, but affected significantly by the response jitter $T_{R}$. Most packet losses are from responses because, for dissemination, each node can receive from not only its parent but also its neighbors. The results show how critical the response synchronization effect is. Finally, Figure 6c shows that Trickle’s suppression threshold *K* does have notable effect on performance, and any value below 5 has insufficient dissemination PRR. Therefore, for the remainder of our evaluation, we use fixed *K* = 5 and $T_{C}$ = 200 ms, with varying $T_{R}$.

### 5.3. Simulation on Various Topology

**Figure 7:**
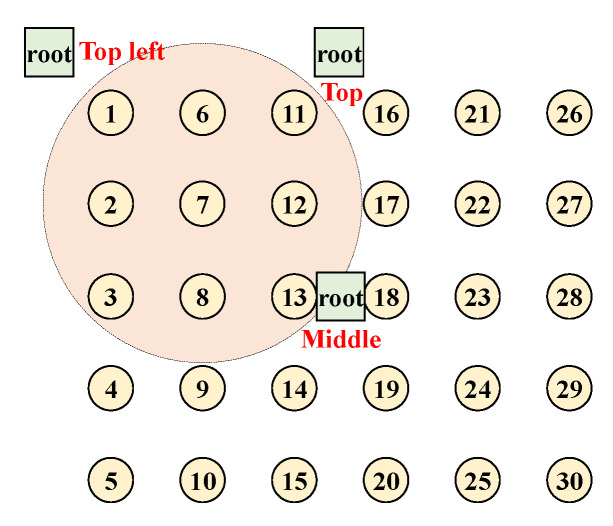
Three-simulation topology with change of root’s position: top-left, top, and middle.

We run simulations on Cooja [40] with four different topologies to compare the three algorithms and see the impact of routing topology. For the first three, nodes are deployed in a grid manner, and only the root’s position is varied to generate different routing topologies (Figure 7).

Last one is the random topology scenario where random topologies are generated by Cooja simulator.

**Figure 8:**
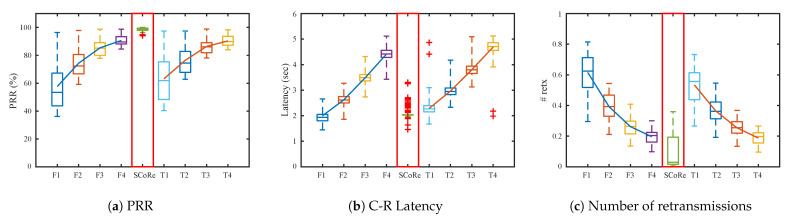
Simulation results when the root is placed at the top-left corner of the grid topology with *M* = 3, *T_C_* = 200 ms, *K* = 5 and varying *T_R_*.

Figure 8 presents the results when the root is deployed on the top-left corner of the deployment. The routing tree on this topology has the longest hop count. Therefore, total amount of traffic in the network is the largest due to forwarding, and results in more link congestion and queue losses. In the figures, *F* stands for “Flooding + RPL-collection” and *T* is “Trickle + RPL-collection”. The numbers after each letter denotes response random jitter $T_{R}$. PRR and the number of packet retransmissions becomes better as the $T_{R}$ increases in both *F* and *T* cases, from 50% to 90%. However, increasing $T_{R}$ causes long network latency as shown in Figure 8b.

On the other hand, SCoRe achieves 99% PRR while keeping the latency near 2 s, only about 7% packets exceed 2 s among 1000 packets. Furthermore, SCoRe has the least number of packet retransmissions compared to others. The few losses come from occasional $N_{\mathrm{dslot}}$ inconsistency and collisions with routing control packets such as DIO and DAO. From the results, we see that SCoRe improves PRR and latency significantly compared to legacy schemes.

**Figure 9:**
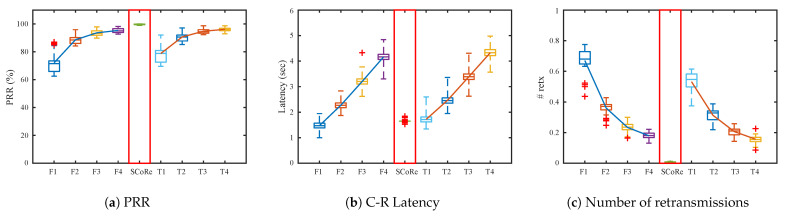
Simulation results when the root is placed in the middle of the grid topology with *M* = 3, *T_C_* = 200 ms, *K* = 5, and varying *T_R_*.

Figure 9 plots the result of simulation with the root at the center of the deployment. Because the network depth is smaller than top-left scenario, all algorithms have better performance than the previous topology. SCoRe can achieve 99% PRR, 1.5 s latency, and the number of response retransmissions decreases close to 0.

**Figure 10:**
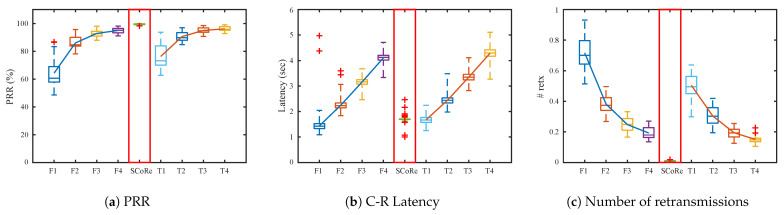
Simulation results from random topologies with *M* = 3, *T_C_* = 200 ms, *K* = 5, and varying *T_R_*.

Figure 10 is the result from random topology scenario. Because distance between nodes in the topology is longer than grid topology, the performance of each algorithm is better overall including SCoRe. SCoRe still keeps 99% PRR and 2 s average latency, but the number of retransmissions decreases evidently. From the result we can see that SCoRe adapts well to other topology, and can expect to have consistent performance in real-world deployments as well.

### 5.4. Testbed Experiment

As a final evaluation, testbed experiments are carried out to verify SCoRe’s performance on real embedded devices. There are numerous reasons that may incur packet loss in real wireless environments such as multipath fading, CTI [53,54], etc., and therefore it is essential to evaluate a wireless protocol through real experiments. For this purpose, we configured an LLN testbed in an office environment with 30 TelosB devices [32] and one gateway, deployed in a grid formation on the ceiling of our lab as shown in Figure 7, where the root is placed at the “Top” position.

**Figure 11:**
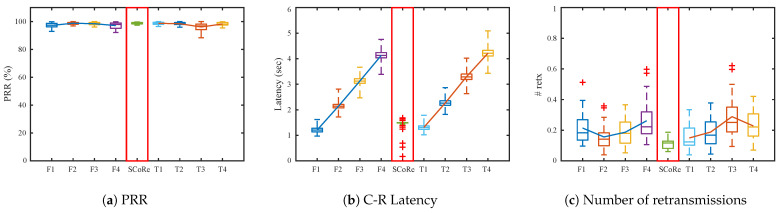
Results from the testbed experiments with *M* = 3, *T_C_* = 200 ms, *K* = 5, and varying *T_R_*.

The experimental results are presented in Figure 11. Because the office room is relatively small, all nodes can receive commands within 1∼2 hops. Therefore, PRR and latency is good enough even when $T_{R}$ is set to 1 second. However, the number of retransmissions for legacy schemes are poor because the response packets are transmitted within a short time window on a highly congested channel in an unscheduled manner. On the other hand, SCoRe’s retransmission count is still close to 0.1 which means SCoRe successfully avoids inter-packet collisions by scheduling command and response jointly. Reduction in retransmission count not only implies higher reliability but also means energy savings. Overall, both the simulation and experiment results show that SCoRe adapts to the network topology while reducing network latency and improving PRR with little number of retransmissions.

## 6. Discussion and Future Work

Although SCoRe suites well for our application needs and system scenarios, it may not be directly applicable to other scenarios. In this section, we discuss the potential limitations of SCoRe and its future work—some ideas to improve SCoRe’s performance even further.

### 6.1. Packet Fragmentation

Because SCoRe piggybacks timeslot information into existing packets, extra room is obviously necessary. As $N_{\mathrm{dslot}}$ is aggregated at each hop, the overhead for $N_{\mathrm{dslot}}$ gathering is fixed and small (2 bytes in our prototype). However, for $N_{\mathrm{dslot}}$ assignment, the scheduling information embedded by each node recursively into command packets is proportional to the number of its children. Because link protocol (e.g., IEEE 802.15.4) has a limit on payload size (e.g., 127 bytes), packet fragmentation technique (e.g., 6LoWPAN) may be necessary to overcome this limitation if we have a larger network.

### 6.2. Coping with Packet Losses

Although collisions between command-phase and response-phase packets are completely eliminated by SCoRe’s relative scheduling, there are various other causes that may incur a packet loss. (e.g., colliding with routing control packet, CTI problems, multipath fading, etc.) Losses trigger link retransmissions, and the retransmissions may trespass on other’s timeslots as they are not accounted for in the schedule. In addition, SCoRe’s assigned slot information is built by the root of each subtree, and carried into command messages. Therefore, each SCoRe node can only accept the command message if and only if the sender is its parent. In other words, a node cannot receive command packets from nodes other than its parent even though it can hear it from other neighbors. If a command packet from its parent is lost, then the descendants of the node will never receive the command. Therefore, this command reception restriction of SCoRe can lead to performance degradation.

Potential future improvements to address these challenges are as follows.

**Recurrent slot assignment.** When a SCoRe root generates a command message, it can set a recurrent bit and omit the scheduling info if the routing topology and $N_{\mathrm{dslot}}$ information has not changed since last command dissemination. A node receiving this command can use timeslots in the same way as the previous command. This method enables each node to be able to receive a command not only from its parent but also neighbors, and thus reduces overhead and improves downward PRR. As a result, recurrent slot assignment makes SCoRe more efficient.
**ETX based timeslot.** A SCoRe node demands *H*, its hop count, for its own response packet transmissions since this is the number of transmissions required to reach the root assuming 100% successful link PRR. However, link retransmissions due to losses may extend beyond its assigned slot, resulting in invading and violating other’s timeslots which will again cause packet collisions. Therefore, careful estimation of the number of retransmissions can help SCoRe to avoid such collisions. ETX [55], expected transmission count, is a very well-known network metric, and RPL also supports ETX based routing called ETXOF [56]. SCoRe can use this metric to request and allocate $N_{\mathrm{dslot}}$ rather than hop count.
**Permeate into lower-layer protocol.** SCoRe’s packet can collide with other protocol’s packets because its on-demand scheduling accounts for only the commands and responses within SCoRe protocol without considering, for example, routing control packets. In fact, most of SCoRe’s packet losses in our evaluations come from packet collisions with RPL routing protocol, the DIO and DAO packets. To avoid this collision, SCoRe may be implemented “jointly” together with the lower-layer protocols. For example, SCoRe can reserve an extra slot within its schedule for other control messages (such as routing) to use. We leave this as our future work.

## 7. Conclusions

SCoRe schedules command and responses jointly, on-demand, in order to improve reliability and latency of the network with little overhead. This is necessary for real-world IoT applications that disseminates commands and collect responses over wireless multihop network in order to control and gather data from multiple embedded devices deployed in the field of interest. Our work was motivated by the fact that most low-power wireless network protocols for dissemination and collection have been designed separately, resulting in severe collisions when used together. Our evaluation results show that SCoRe improves reliability and latency simultaneously, and this was achieved dynamically at runtime without a preconfigured slot assignments nor time synchronization nor tuning the parameters.
